# T1- Thresholds in Black Holes Increase Clinical-Radiological Correlation in Multiple Sclerosis Patients

**DOI:** 10.1371/journal.pone.0144693

**Published:** 2015-12-11

**Authors:** Christian Thaler, Tobias Faizy, Jan Sedlacik, Brigitte Holst, Jan-Patrick Stellmann, Kim Lea Young, Christoph Heesen, Jens Fiehler, Susanne Siemonsen

**Affiliations:** 1 Department of Diagnostic and Interventional Neuroradiology, University Medical Centre Hamburg-Eppendorf, Hamburg, Germany; 2 Department of Neurology, University Medical Centre Hamburg-Eppendorf, Hamburg, Germany; 3 Institute for Neuroimmunology and Clinical MS Research, University Medical Centre Hamburg-Eppendorf, Hamburg, Germany; University of Oxford, UNITED KINGDOM

## Abstract

**Background:**

Magnetic Resonance Imaging (MRI) is an established tool in diagnosing and evaluating disease activity in Multiple Sclerosis (MS). While clinical-radiological correlations are limited in general, hypointense T1 lesions (also known as Black Holes (BH)) have shown some promising results. The definition of BHs is very heterogeneous and depends on subjective visual evaluation.

**Objective:**

We aimed to improve clinical-radiological correlations by defining BHs using T1 relaxation time (T1-RT) thresholds to achieve best possible correlation between BH lesion volume and clinical disability.

**Method:**

40 patients with mainly relapsing-remitting MS underwent MRI including 3-dimensional fluid attenuated inversion recovery (FLAIR), magnetization-prepared rapid gradient echo (MPRAGE) before and after Gadolinium (GD) injection and double inversion-contrast magnetization-prepared rapid gradient echo (MP2RAGE) sequences. BHs (BH_vis_) were marked by two raters on native T1-weighted (T1w)-MPRAGE, contrast-enhancing lesions (CE lesions) on T1w-MPRAGE after GD and FLAIR lesions (total-FLAIR lesions) were detected separately. BH_vis_ and total-FLAIR lesion maps were registered to MP2RAGE images, and the mean T1-RT were calculated for all lesion ROIs. Mean T1 values of the cortex (CTX) were calculated for each patient. Subsequently, Spearman rank correlations between clinical scores (Expanded Disability Status Scale and Multiple Sclerosis Functional Composite) and lesion volume were determined for different T1-RT thresholds.

**Results:**

Significant differences in T1-RT were obtained between all different lesion types with highest T1 values in visually marked BHs (BH_vis_: 1453.3±213.4 ms, total-FLAIR lesions: 1394.33±187.38 ms, CTX: 1305.6±35.8 ms; p<0.05). Significant correlations between BH_vis_/total-FLAIR lesion volume and clinical disability were obtained for a wide range of T1-RT thresholds. The highest correlation for BH_vis_ and total-FLAIR lesion masks were found at T1-RT>1500 ms (Expanded Disability Status Scale vs. lesion volume: r_BHvis_ = 0.442 and r_***total-FLAIR***_ = 0.497, p<0.05; Multiple Sclerosis Functional Composite vs. lesion volume: r_BHvis_ = -0.53 and r_***total-FLAIR***_ = -0.627, p<0.05).

**Conclusion:**

Clinical-radiological correlations in MS patients are increased by application of T1-RT thresholds. With the short acquisition time of the MP2RAGE sequences, quantitative T1 maps could be easily established in clinical studies.

## Introduction

Multiple sclerosis (MS) is a chronic inflammatory disease of the central nervous system leading to demyelination, axonal loss and the formation of astrocytic scars [1]. Magnetic resonance imaging (MRI) is a valuable tool for diagnosis and monitoring disease activity in MS.[2,3] White matter lesions can be detected in T2-weighted (T2w) images as areas of high signal intensity. Hyperintensities on T2w images correspond to a wide spectrum of histopathological changes, ranging from edema and mild demyelination to glial scars or liquid necrosis. [4,5,6]. In comparison, persistent non-enhancing T1-hypointense lesions (commonly known as black holes (BH)) are discussed as a more specific marker for demyelination, axonal loss and tissue damage[7,8] and are thought to provide a more accurate classification of the microstructural damage in MS patients. However, histopathological studies demonstrated that even BHs themselves present pathological diversity.[9] To discriminate the different stages of cell damage it was observed that the degree of hypointensity in a BH seems to reflect the extent of axonal loss and therefore might distinguish between demyelinated and partially remyelinated lesions.[10,11] Nevertheless, the definition of BHs is very heterogeneous and in general depends on visual evaluation.

It is still uncertain whether BHs correlate with the patients' clinical status, since some research groups could not find any significant correlation but others did.[12–18] A number of studies suggested that lesion intensity or T1 relaxation time (T1-RT) might be useful to increase the correlation between BH volume and clinical measures.[16,18] In a study by Tam et al. (2011), a T1 lesion load was computed at different signal intensity thresholds and the resulting lesion volumes at a given intensity level were correlated with the Expanded Disability Status Scale (EDSS).[16] The strongest correlations were found by limiting the BH regions to a small subset of the voxels with lowest signal intensity. However, scan-to-scan variations affect the quantitative signal intensity, which reduces the reliability of the measurements. To minimize scan-to-scan variations, quantitative T1-RT mapping has been found to increase accuracy and reproducibility.[19] With the recently introduced double inversion-contrast magnetization-prepared rapid gradient echo (MP2RAGE) sequence it is now possible to generate T1-RT maps in clinically acceptable scan times on the fly.[20] We hypothesized that higher correlation coefficients for BH lesion volume and clinical disability can be achieved by applying T1-RT thresholds. To our knowledge, this is the first study investigating the range of correlation coefficients for BH volumes and EDSS as well as the MSFC at multiple T1-RT thresholds.

## Materials and Methods

Forty patients (26 females, 14 males; 36.9 ± 10.6 years) with mainly relapsing-remitting multiple sclerosis (RRMS) were enrolled in this study. One patient was diagnosed with primary-progressive (PPMS) and two with secondary-progressive multiple sclerosis (SPMS). Disease duration ranged from 1 month up to 22 years. Clinical status was assessed by using the EDSS by experienced neurologists according to published guidelines.[21] The Multiple Sclerosis Functional Composite (MSFC) was additionally assessed at 30 patients and included the Symbol Digit Modalities Test (SDMT), the 9-Hole Peg Test (9-HPT) and the Timed 25-Foot Walk (T25-FW). Within the MSFC the Paced Auditory Serial Additon Test (PASAT) was substituded by the SDMT based on higher patient acceptance and at least equal sensitivity.[22] Scores for each subtest were converted to z-scores using a published dataset.[23] Subsequently, the overall MSFC score was calculated with following formula: MSFC Score = {Z_9-HPT_+Z_T25-FW_+Z_SDMT_}/3. The patient characteristics are summarized in Table 1. The study was approved by the local Ethical Committee Hamburg (Ethik-Kommission der Ärztekammer Hamburg) following the guidelines of the Declaration of Helsinki and patients provided written informed consent.

**Table 1:** Subject population. Age (years), EDSS, disease duration (years), gender and MS-type statistics for the 40 patients in this study.

	mean	SD	range (median)
Age	36.6	10.6	18–64 (36.5)
EDSS	2.2	* 1.5	0–6.5 (2)
MSFC*	0.98	1.14	-1.86–2.99 (1.01)
Disease Duration	6.8	6.5	0–22 (4)
Gender	26 females, 14 males		
MS-type	37 RRMS, 2 SPMS, 1 PPMS		

*n = 30

EDSS = Expanded Disability Status Scale, SD = standard deviation, RRMS = relapsing-remitting multiple sclerosis, SPMS = secondary-progressive multiple sclerosis, PPMS = primary-progressive multiple sclerosis

### MRI data acquisition

All MR scans were performed on a 3 Tesla MR scanner (Skyra, Siemens Medical Systems, Erlangen, Germany). The MR protocol included a sagittal 3-dimensional fluid attenuated inversion recovery (FLAIR) (echo-time (TE) = 390 ms, repetition time (TR) = 4700 ms, inversion time (TI) = 1800 ms, 192 slices, field of view (FOV) = 256 mm, voxel size = 1.0 x 1.0 x 1.0 mm), a T1-weighted magnetization-prepared rapid gradient echo (MPRAGE) sequence before and after Gadolinium injection (TE = 2.43 ms, TR = 1900 ms, TI = 900 ms, 192 slices, FOV = 256 mm, voxel size = 1.0 x 1.0 x 1.0 mm, flip-angle = 9°) and MP2RAGE (TE = 2.98 ms, TR = 5000 ms, TI = 700 ms, 176 slices, FOV = 256 mm, voxel size = 1.0 x 1.0 x 1.0 mm, flip angle = 4°). Gadolinium was applied to detect enhancing lesions, which were excluded in the volumetric calculations of BHs_vis_. The MP2RAGE sequence acquisition time was 11 minutes and 17 seconds.

### Image analysis

BHs were defined as non-enhancing lesions that appear hypointense on T1w-images with signal intensity below cortex and are concordant with hyperintense lesions on a T2w-image. BH-lesion detection was performed by two independent raters who were blinded to the patients' clinical status. For each patient, T1 hypointense lesions were outlined on T1w images according to the definition above by both raters using the software Analyze 11.0 (AnalyzeDirect, Inc. KS, USA). The resulting BH lesion masks of both raters were binarized and patient-wise multiplied to obtain a consensus mask. Subsequently, T1w-MPRAGE images were linearly registered to the MP2RAGE images and the transformation applied to the BH consensus masks. We will refer to these manually segmented BH as visually marked BHs (BH_vis_). In a second step, contrast enhancing (CE-lesions) were marked applying the same algorithm as described for BH lesion outlining.

**Fig 1:**
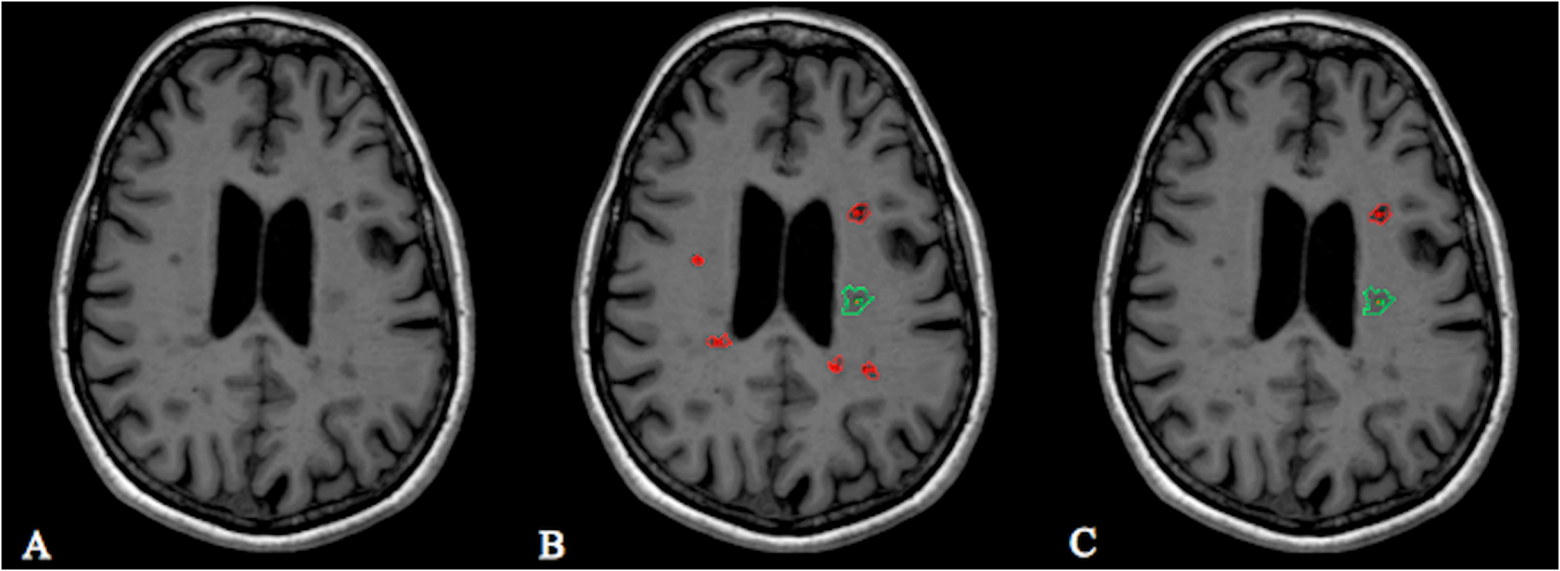
Example of ROI volumes overlaid on T1w images before and after setting a T1-RT threshold. **A**: Axial slice of the cerebrum with multiple T1 hypointense lesions. **B**: Manually placed ROIs without applied threshold: BH_vis_ (red), CE lesion (green). **C: Remaining ROIs after applying the obtained optimal threshold of T1-RT >1500 ms with highest correlation to EDDS; CE lesions were not affected by thresholds.**

Additionally, a second lesion segmentation was performed, by marking hyperintense lesions in FLAIR images. As every BH_vis_ appears hyperintense in FLAIR images, the BHs_vis_ are element of the FLAIR lesions. FLAIR lesion masks (total-FLAIR) were created by using an open source lesion segmentation software (LST: Lesion Segmentation Tool).[24] To choose the optimal initial threshold κ, lesion segmentation was run with different thresholds. The final threshold was set at κ = 0.3. The semi-automatically segmented total-FLAIR lesion masks were visually controlled and corrected if necessary. Afterwards the lesion masks were registered to the MP2RAGE images. In addition, a pure-FLAIR lesion mask was calculated by subtracting the BH_vis_ and CE lesion mask from the corresponding total-FLAIR mask leaving only those lesions, that were visible in FLAIR but did not correspond to a BH_vis_ or CE lesion. For each patient, total-FLAIR, pure-FLAIR, CE and BH_vis_ lesion number, volume and mean T1-RT were calculated. In addition, voxel-wise T1-RT values were obtained for each lesion group. Furthermore, mean T1-RT values of the cortex were calculated for each patient by manually placing region of interests (ROIs) in the frontal, parietal, temporal and occipital cortex. BH_vis_ and total-FLAIR lesion volumes were also computed at eleven different T1-RT thresholds including all lesions (BH_thr_, total-FLAIR_thr_) with a mean T1-RT above the given threshold (Fig 1). T1-RT thresholds were ranging from 700 ms to 1700 ms.

### Statistical analysis

Statistical analysis was performed by using Statistics in R 3.0.0 and IBM SPSS 21.0. Interrater reliability for BH_vis_ lesions masks was quantified using the Dice overlap coefficient. T1-RTs in the different lesion groups were compared using unpaired two-samples t-test. Spearman rank correlations between EDSS and voxel-/lesion volume were determined for different T1-RT thresholds. Voxel and lesion volumes were computed for BH_vis_ lesion masks as well as for total-FLAIR and pure-FLAIR lesion masks.

## Results

### Interrater Reliability

Because of the diverse definitions of BHs and the heterogeneous presentation of T1w hypointense lesions, a Dice overlap coefficient was generated to quantify interrater reliability. When comparing the BH_vis_ masks of both raters from all patients, an overall Dice coefficient of 0.8 was calculated.

### Quantitative T1 values

**Fig 2:**
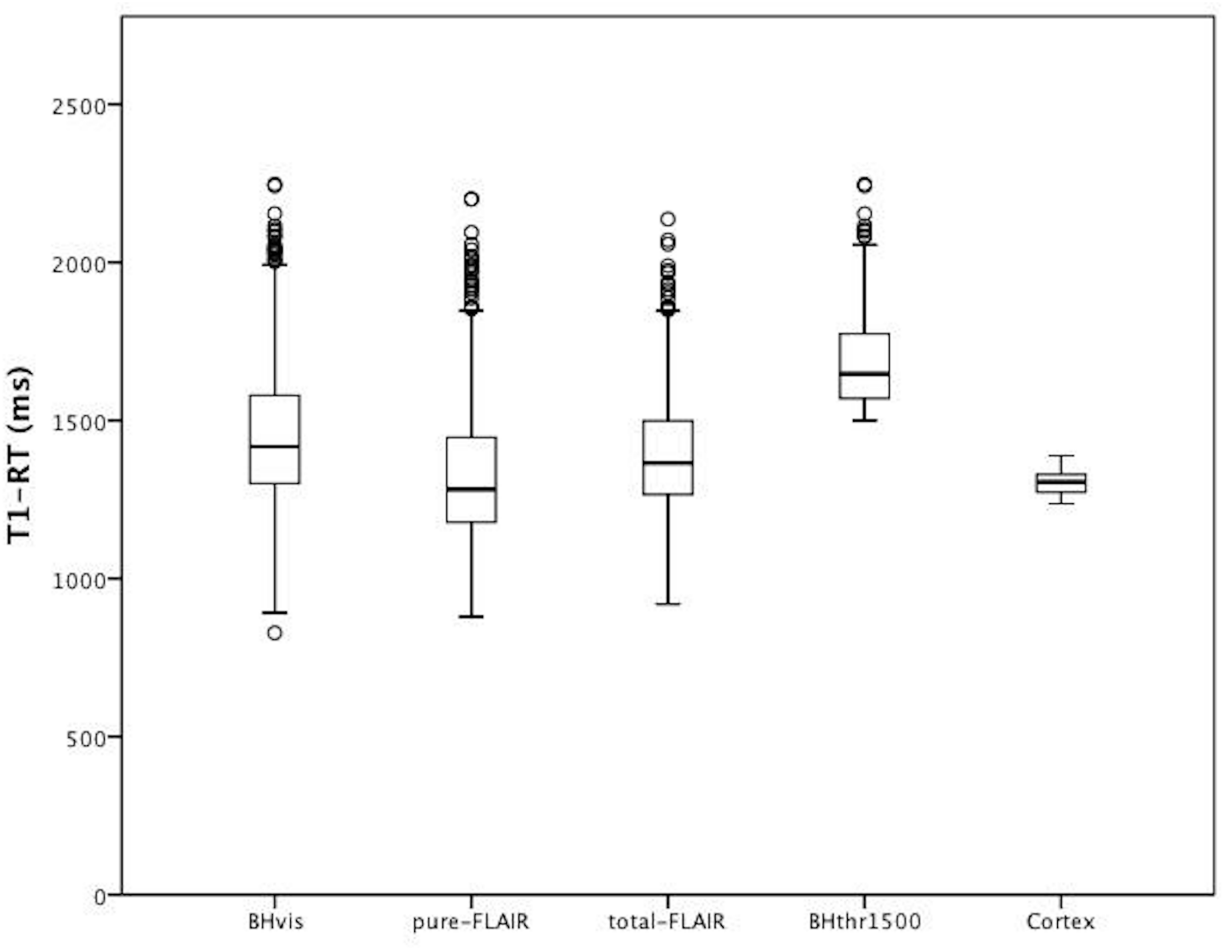
Box Plots showing T1-RT distribution in different lesion types. T1-RT = T1 relaxation time; total-FLAIR lesions = lesions from the semi-automatically segmented FLAIR lesions masks; pure-FLAIR lesions = lesions from the semi-automatically segmented FLAIR lesions masks that did not correspond to a BH_vis_ or CE lesion; BH_vis_ = manually segmented black holes without applied threshold, BHs_thr1500_ = manually segmented black holes with T1-RT higher than 1500 ms.

For every patient, BH_vis,_ pure-FLAIR and total-FLAIR lesion numbers, volumes and mean T1-RT were computed and are displayed in Table 2. There were significant differences in T1-RT between all lesion groups (p<0.05) with highest T1-RT values in BHs_vis_, which was also the lesion group with the largest standard deviation. The mean BH_vis_ number exceeded the mean total-FLAIR lesion number, which is caused by the procedure that only voxels with intensity lower than cortex were marked as BHs_vis_ in T1w images, so that in some cases one large FLAIR lesion appeared as multiple smaller BHs_vis_. CE lesions were detected in 15 patients. The T1-RT measured in the cortex was very uniform with the lowest standard deviation (1305.6 ± 35.8 ms). The mean T1-RT in BHs_vis_ was significantly lower than T1-RT values measured in the cortex (T1-RT_BHvis_ = 1453.3 ± 213.4 ms vs. T1-RT_Cortex_ = 1305.6 ± 35.8 ms; p<0.05). Fig 2 **presents T1-RT in the different lesion types.**

**Table 2:** Lesion mask characteristics. Mean number, volume and T1-RT for different lesion classifications and cortex.

	total-FLAIR lesions	pure-FLAIR lesions	BHs_vis_	BHs_thr1500_	Cortex
Number	19.83 ± 15.85	19.95 ± 17.18	22.3 ± 17.07	9.18 ± 8.24	-
Volume [mm^3^]	146.26 ± 326.36	98.75 ± 239.97	82.4 ± 128	126.83 ± 178.84	-
T1-RT [ms]	1394.33 ± 187.38	1331.19 ± 226.78	1453.3 ± 213.4	1688.7 ± 154.26	1305.6 ± 35.8

T1-RT = T1 relaxation time; total-FLAIR lesions = lesions from the semi-automatically segmented FLAIR lesions masks; pure-FLAIR lesions = lesions from the semi-automatically segmented FLAIR lesions masks that did not correspond to a BH_vis_ or CE lesion; BH_vis_ = manually segmented black holes without applied threshold, BHs_thr1500_ = manually segmented black holes with T1-RT higher than 1500 ms

### Spearman rank correlations

#### Lesion volume and Expanded Disability Status Scale

**Fig 3:**
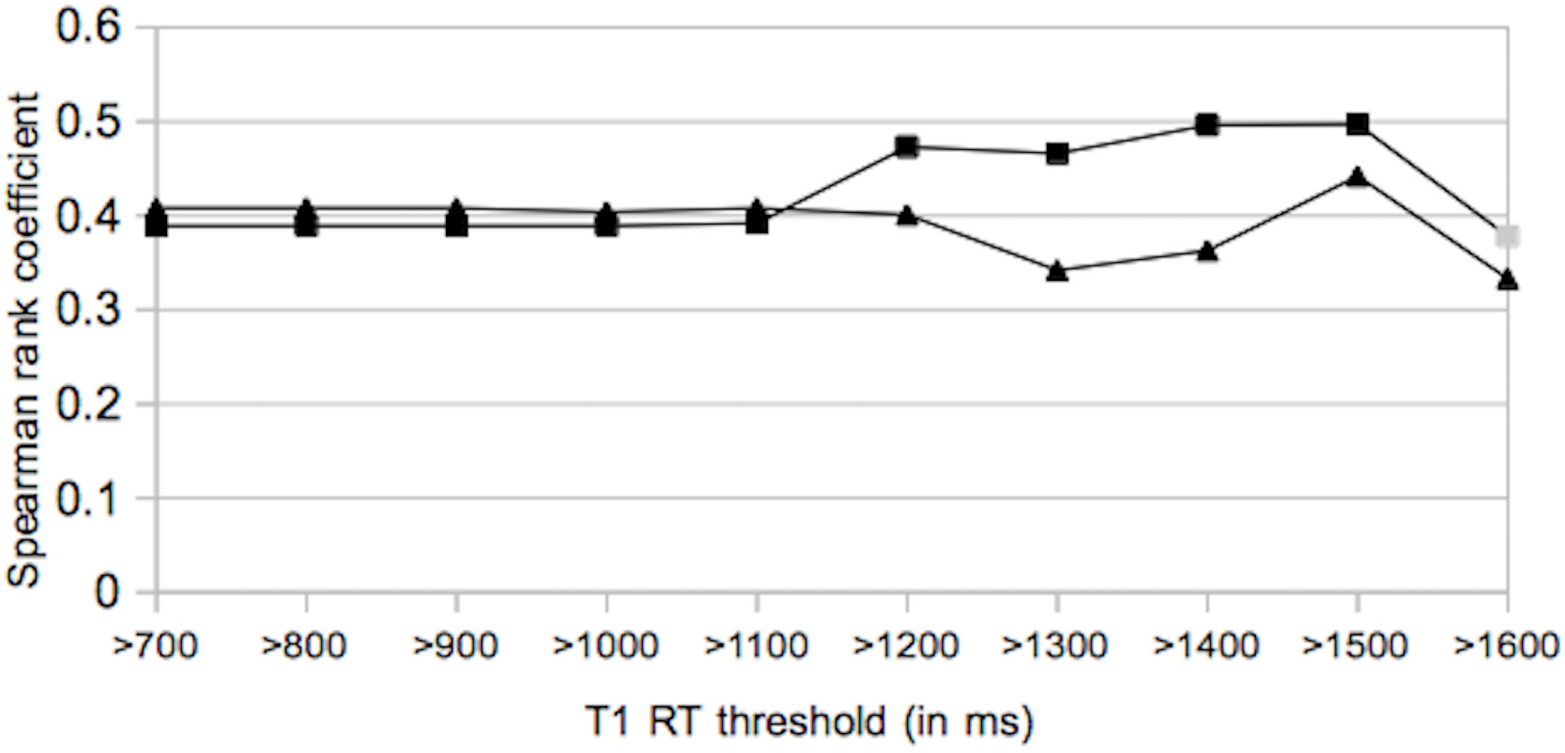
Spearman rank correlation coefficients for lesion volume and EDSS at different T1-RT thresholds. ▲ = BHs_thr_ volume vs. EDSS, ◙ = total-FLAIR_thr_ lesion volume vs. EDSS (black symbols indicate a significant correlation). T1-RT = T1 relaxation time.

Spearman rank correlation coefficients for BH_thr_ volume and EDSS as well as total-FLAIR_thr_ lesion volume and EDSS were calculated for eleven different T1-RT thresholds ranging from 700 ms to 1700 ms. A T1-RT threshold of 700 ms included all BHs_vis_ and total-FLAIR lesions and similar correlation coefficients were obtained (r_BHthr_ = 0.408, r_total-FLAIRthr_ = 0.389). For BHs_thr_, correlation showed constant values until it decreases to r_BHthr_ = 0.342 at a T1-RT threshold of 1300 ms. Maximum correlation of r_BHthr_ = 0.442 was obtained by including only lesions with mean T1-RT >1500 ms. A similar trend was observed for ROIs of the total-FLAIR lesion masks. As T1-RT thresholds increase, only hypointense lesions in MP2RAGE images remain. Starting at a minimum of r_total-FLAIRthr_ = 0.389 at a T1-RT of 700 ms, the correlations increased to a peak of r_total-FLAIRthr_ = 0.497 at a T1-RT threshold of 1500 ms. Regarding BHs_thr,_ no significant correlations were found for T1-RT >1700 ms or at higher thresholds. For total-FLAIR_thr_ lesions, no significant correlations were found for T1-RT >1600 ms or higher. At the optimal T1-RT threshold of 1500 ms 21% of the initial total-FLAIR_thr_ lesion volume and 54% of the initial BH_vis_ volume remain. Table 3 and Fig 3 present the computed correlation coefficients. In comparison, volumes determined from the application of voxel-wise thresholds did not increase correlation coefficients.

**Table 3:** Spearman rank correlations coefficients for lesion volume and EDSS at different T1-RT thresholds. T1-TR thresholds in ms, BH_thr_ volume = mean cumulative BH_thr_ volume per patient in mm^3^, total-FLAIR_thr_ lesion volume = mean cumulative total-FLAIR_thr_ lesion volume per patient in mm^3^, Spearman r = Spearman rank coefficients for lesion volume and EDSS.

T1-RT threshold	BH_thr_ volume	Spearman r	p	total-FLAIR_thr_ lesion volume	Spearman r	p
>700	1836.78	0.408	**0.009**	2964.62	0.389	**0.007**
>800	1836.78	0.408	**0.009**	2964.62	0.389	**0.007**
>900	1836.43	0.408	**0.004**	2964.62	0.389	**0.007**
>1000	1836.05	0.404	**0.005**	2964.47	0.389	**0.007**
>1100	1832.33	0.408	**0.004**	2945.4	0.392	**0.006**
>1200	1797.18	0.401	**0.005**	2910.62	0.473	**0.001**
>1300	1702.16	0.342	**0.018**	2557.96	0.466	**0.003**
>1400	1436.65	0.363	**0.014**	1748.42	0.496	**0.002**
>1500	1163.83	0.442	**0.004**	973.67	0.497	**0.006**
>1600	971.19	0.333	**0.039**	362.17	0.378	0.05

T1-RT = T1 relaxation time, BH_thr_ = manually segmented black holes after thresholding, BH_vis_ = manually segmented black holes without applied threshold, total-FLAIR_thr_ lesions = lesions from the semi-automatically segmented FLAIR lesions masks after thresholding, total-FLAIR = lesions from the semi-automatically segmented FLAIR lesions masks without applied threshold

#### Lesion volume and Multiple Sclerosis Functional Composite

**Fig 4:**
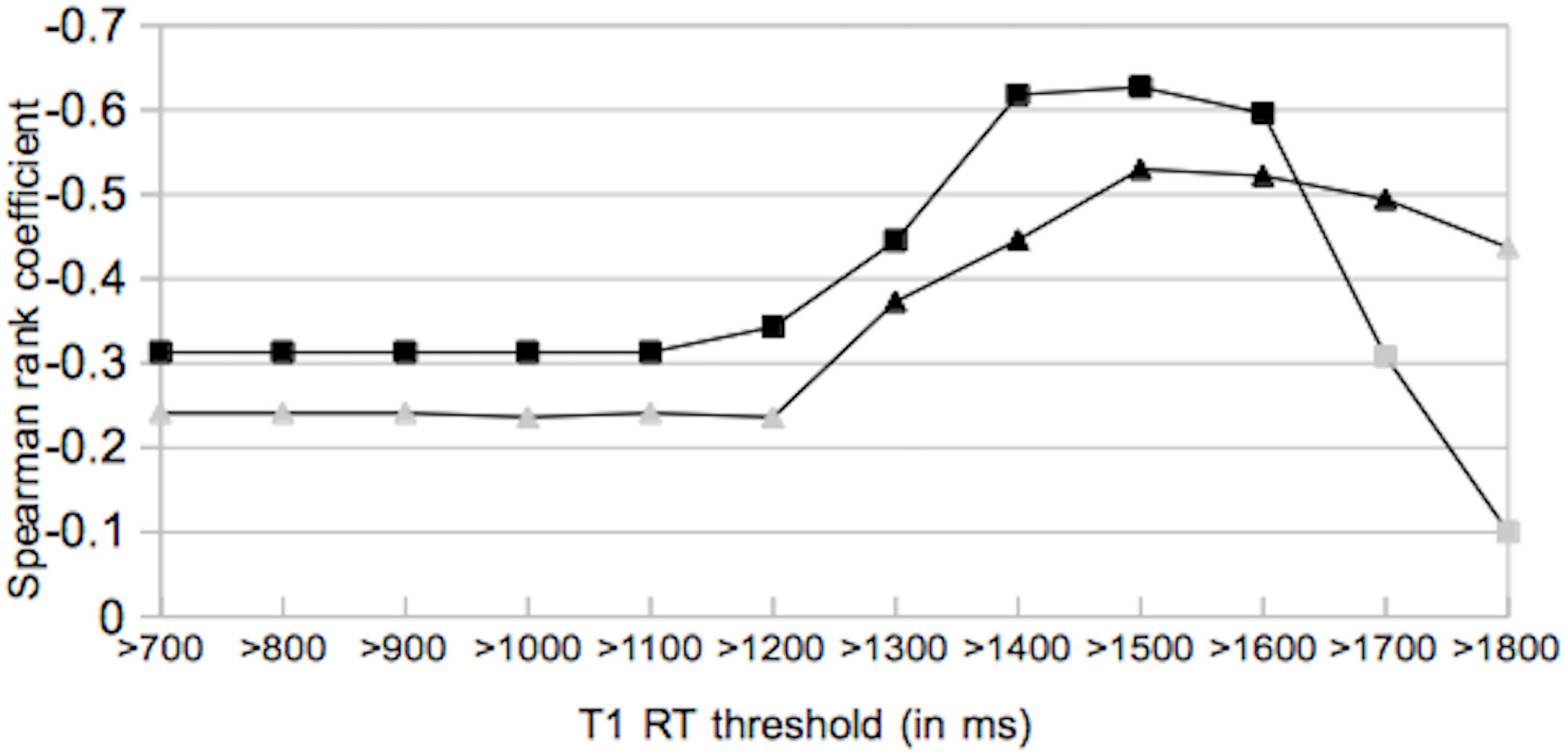
Spearman rank correlation coefficients for lesion volume and MSFC at different T1-RT thresholds. ▲ = BHs_thr_ volume vs. MSFC, ◙ = total-FLAIR_thr_ lesion volume vs. MSFC (black symbols indicate a significant correlation). T1-RT = T1 relaxation time.

In 30 patients Spearman rank correlation coefficients for lesion volumes and MSFC were obtained for twelve different T1-RT thresholds ranging from 700 to 1800 ms. At a T1-RT threshold of 700 ms a correlation coefficient of -0.313 was obtained for total-FLAIR lesion volume and MSFC. As thresholds raise also correlation coefficients for total-FLAIR lesion volume and MSFC show increasing values until maximum correlation is achieved at T1-RT>1500 ms (r_total-FLAIRthr_ = -0.627). For BH_thr_ no significant correlations were found for T1-RT threshold between 700 and 1200 ms. At 1300 ms a significant correlation coefficient of -0.373 is obtained. Correlations coeffecients increase to a peak of r_BHthr_ = -0.53 at a T1-RT threshold of 1500 ms. A detailed overview is presented in Table 4 and Fig 4.

**Table 4:** Spearman rank correlations coefficients for lesion volume and MSFC at different T1-RT thresholds. T1-TR thresholds in ms, BH_thr_ volume = mean cumulative BH_thr_ volume per patient in mm^3^, total-FLAIR_thr_ lesion volume = mean cumulative total-FLAIR_thr_ lesion volume per patient in mm^3^, Spearman r = Spearman rank coefficients for lesion volume and EDSS.

T1-RT threshold	BH_thr_ volume	Spearman r	p	total-FLAIR_thr_ lesion volume	Spearman r	p
>700	1651.97	-0.241	0.1	2713.88	-0.313	**0.046**
>800	1651.97	-0.241	0.1	2713.88	-0.313	**0.046**
>900	1651.87	-0.241	0.1	2713.88	-0.313	**0.046**
>1000	1651.37	-0.236	0.105	2713.68	-0.313	**0.046**
>1100	1650	-0.241	0.1	2693.25	-0.313	**0.046**
>1200	1616.68	-0.236	0.105	2560.96	-0.343	**0.034**
>1300	1533.99	-0.373	**0.025**	2197.63	-0.445	**0.011**
>1400	1242.38	0.446	**0.009**	1495.82	-0.618	**0.001**
>1500	954.6	-0.53	**0.003**	1084.72	-0.627	**0.004**
>1600	834.33	-0.522	**0.006**	358.11	-0.596	**0.012**
>1700	735.05	-0.494	**0.026**	277.81	-0.308	0.165
>1800	594.73	-0.437	0.059	172.76	-0.1	0.399

T1-RT = T1 relaxation time, BH_thr_ = manually segmented black holes after thresholding, BH_vis_ = manually segmented black holes without applied threshold, total-FLAIR_thr_ lesions = lesions from the semi-automatically segmented FLAIR lesions masks after thresholding, total-FLAIR = lesions from the semi-automatically segmented FLAIR lesions masks without applied threshold

The highest correlation was found at T1-RT >1500 ms for both lesion masks. Altogether, by using the total-FLAIR lesion masks as basis for successive thresholding, slightly higher correlation coefficients are presented between lesion volumes and EDSS as well as MSFC. Regarding BHs_thr_, two patients of our study group had to be excluded at the optimal threshold of 1500 ms because they did not show lesions with T1-RT values below signal intensity. By using the total-FLAIR_thr_ lesion masks 15 patients were excluded at given threshold.

Correlation coefficients were also obtained for voxel-wise instead of lesion-wise analyses. Since all patients displayed lesions containing voxels above the applied thresholds, no patient was excluded for voxel-wise analysis. The resulting correlation coefficients showed no meaningful difference in comparison to correlation coefficients obtained from the corresponding lesion-wise analysis.

We also found a significant association for EDSS and lesion number but with decreased correlation coefficients. For BHs_thr_ the highest correlation was found at T1-RT >1500 ms with a Spearman correlation coefficient of 0.352 (p = 0.02) and for total-FLAIR_thr_ lesions the highest correlation was found at T1-RT >1400 ms with a Spearman correlation coefficient of 0.476 (p = 0.003).

Correlation analyses were als perfomed for number of black holes and disease duration (Spearman rank coefficient of 0.422; p = 0.003) as well as for cumulative black hole volume and disease duration (Spearman rank coefficient of 0.433; p = 0.003). After applying T1 RT thresholds only minor changes in Spearman rank coefficients were observed for lesion volume and disease duration

There was no significant correlation between EDSS or MSFC and disease duration in our study cohort.

## Discussion

The aim of this study was to assess the influence of T1-RT variations on the correlation between BH lesion volume and clinical disability. Our results suggest, that T1-RT has a significant influence on clinical-radiological correlations and may improve the differentiation of T1w hypointense lesions. T1-RT helps to provide further information on pathological changes in T1 hypointense lesions, indicating the degree of tissue damage within the observed region, such as demyelination, axonal loss and gliosis.[7,9,25–27] It has already been observed in previous studies, that T1-RT differs significantly in normal appearing white matter in patients with RRMS and SPMS compared with healthy controls.[28,29] However, only few studies exist focusing on T1-RT in BHs and its impact on clinical-radiological correlation. Just recently Simioni et al. (2014) reported a relation between increased T1-RT in T1 hypointense lesions and cognitive performance in MS patients but no correlation with other clinical deficits was performed.[18]

In our patient group, the strongest correlation between lesion volume and clinical scores, measured by EDSS and MSFC, was observed at a T1-RT threshold of 1500 ms for lesions remaining in the BH_vis_ masks as well as for lesions remaining in the total-FLAIR lesion masks. Regarding BH_vis_ lesion volume and MSFC, significant correlations were only achieved after applying thresholds. Similar results were presented by Tam et al. (2011), showing that the strongest correlation was achieved by only including the darkest voxels in T1 lesion masks.[16] Unlike Tam et al. (2011) we didn't measure relative intensities from T1-weighted images but quantitative T1 values. Thus, scan-to-scan variations were minimized and a higher reliability was achieved. Our results show that the best clinical correlation was achieved by only including lesions with T1-RT clearly higher than cortex, indicating high severity of tissue damage.[9]

We found similar correlation coefficients by using the manually processed BH_vis_ masks and the semi-automatically segmented total-FLAIR lesion masks. It is not surprising that correlation coefficients derived from thresholding both of these masks have their peak at T1-RT = 1500 ms, as the BH_vis_ mask is element of the total-FLAIR lesion mask, so that T1 hypointense lesions with the darkest voxels remaining in both masks can be expected to largely overlap. However, higher correlation coefficients were obtained by using the total-FLAIR lesion masks as a basis for thresholding. A reason for this observation might be, that the manual BH lesion segmentation suffers from observer variability, which might lead to decreased objectivity and sensitivity corresponding to voxels below the applied threshold that might have been missed as BH-voxels. Also there is no uniform definition of BHs as some authors define them as lesions on T1-weighted images with lower signal intensity compared with surrounding normal-appearing white matter[30,31] while others define them as lesions on T1-weighted images with signal intensity below cortex[32,33]. These definitions are therefore not only dependent on the rater but also on sequence parameters. In our study BHs were defined as the latter and mean T1-RT in BHs_vis_ differed significantly from T1-RT measured in total-FLAIR lesions and cortex (T1-RT_BHvis_ = 1453.3 ± 213.4 ms vs. T1-RT_Cortex_ = 1305.6 ± 35.8 ms and T1-RT_total-FLAIR_ = 1394.33 ± 187.38, p < 0.05). As darker voxels seem to have a stronger impact on clinical disability, we suggest defining BHs by T1-RT to increase clinical-radiological correlations and help setting a more objective definition. (Semi-) automatic segmentation might be a useful tool to increase objectivity and establish a more specific definition of BHs.

For semi-automatic lesion segmentation we used the Lesion Segmentation Toolbox, which is implemented in SPM8. LST is a well established segmentation software and widely used in former MS clinical trials.[24,34,35] Moreover, good agreement was found when comparing LST to manual segmentation.[24]

A possible limitation of our study is, that we did not classify lesions by their location, as cortical lesions have been found to contribute to higher levels of physical and cognitive disability.[16,36] Nevertheless, every detected lesion regardless of its location was affected by T1-RT thresholding. Further investigation of the localization of lesions and corresponding T1-RT will be useful for a better understanding of these two important radiologic parameters.

Secondly, we also included patients with CE lesions, which indicate active inflammation and might therefore impair the patients' clinical status. However, controlling for CE lesions did not impact on study results. Therefore we believe correlates are robust in patients with and without CE lesions.

We used the EDSS to measure clinical disability, which is well known for its intrinsic limitation. Beside its bias towards mobility and high inter-observer variability especially in the lower range, subtle clinical progression is often missed by its non-linear rating.[37] However, correlation coefficients were also obtained by using the MSFC, which extends measures of functional status and correlates with EDSS scores in MS patients.[38]

In conclusion, our findings suggest that assessing T1 lesions in patients with Multiple sclerosis by its T1-RTs increases clinical-radiological correlations. With the fast acquisition time of the MP2RAGE sequences, generating quantitative T1 maps can be established in clinical studies. Further emphasis should be laid on longitudinal observation to assess the prognostic value of these measurements.

## Supporting Information

S1 Table Lesion characteristics. (XLS)

S2 Table Patient characteristics. (XLS)
